# Aerosol Dynamics in the Respiratory Tract of Food-Producing Animals: An Insight into Transmission Patterns and Deposition Distribution

**DOI:** 10.3390/ani15101396

**Published:** 2025-05-12

**Authors:** Longhuan Du, Mohan Qiu, Zengrong Zhang, Chenming Hu, Li Yang, Zhuxiang Xiong, Jiangxian Wang, Xia Xiong, Han Peng, Jialei Chen, Shiliang Zhu, Xiaoyan Song, Chunlin Yu, Chaowu Yang

**Affiliations:** 1 Sichuan Animal Science Academy, Chengdu 610066, China; longhuan_du@163.com (L.D.);; 2 Animal Breeding and Genetics Key Laboratory of Sichuan Province, Chengdu 610066, China

**Keywords:** airborne disease, respiratory tract, aerosol dynamics, CT scan, CFD, aerosolized vaccines

## Abstract

Airborne diseases pose a significant challenge in intensive livestock farming due to their rapid transmission. Aerosols facilitate the spread of pathogens, introducing external infections to farms and enabling cross-transmission within barns. To address knowledge gaps in aerosol dynamics in animal respiratory tracts and enhance understanding of airborne disease transmission, this study employed CT scanning, 3D printing, and CFD technologies to develop and validate a pig respiratory model. Qualitative and quantitative results from the present study reveal spatiotemporal heterogeneity in aerosol deposition and transmission. Under rest conditions, for aerosols with D ≤ 5.0 μm, 21.1% of inhaled aerosols were deposited in the lung by the end of a respiratory cycle. Doubling the respiratory cycle or the inhalation rate could further increase the penetration ability of small-sized aerosols by approximately 60% to 70%. Moreover, the asymmetric distribution of airflow between the left and right halves of the lower respiratory tract (*Q_L_*_/*R*_ = 0.89) resulted from the leftward position of the pig’s heart and consequently led to a deposition ratio of about 0.83 between the left and right bronchial airways. These findings provide fundamental scientific data for the development and application of aerosolized vaccines and offer insights into optimizing respiratory intervention strategies.

## Simple Summary

Airborne diseases pose significant challenges in intensive livestock farming due to aerosol-mediated pathogen transmission. This study employed CT scanning, 3D printing, and CFD to investigate aerosol dynamics in porcine respiratory tracts. Results revealed spatiotemporal heterogeneity in aerosol deposition; under resting conditions, 21.1% of inhaled aerosols (D ≤ 5.0 μm) were deposited in the lungs, while 27.5% adhered to airway walls. Doubling the respiratory cycle duration or the inhalation rate enhanced small aerosol penetration by 60–70%, with minimal impact on large particle deposition in the upper airways. Cardiac leftward displacement induced asymmetric airflow distribution (*Q_L_*_/*R*_ = 0.89) in the lower respiratory tract, resulting in a 0.83 deposition ratio between the left and right bronchial airways. These findings provide foundational data for optimizing aerosolized vaccine delivery and respiratory intervention strategies in livestock management.

## 1. Introduction

Airborne disease transmission is a common yet challenging route of infection in livestock farming that is particularly difficult to control due to its rapid and widespread nature [1]. Common airborne diseases that have caused significant economic losses worldwide include African Swine Fever (ASF) [2], Mycoplasmal Pneumonia of Swine (MPS) [3], Swine Streptococcosis [4], Avian Influenza (AI) [5], Newcastle Disease (ND) [6], etc. Once an airborne disease breaks out, it can quickly propagate through animal populations, especially in large-scale, intensive, and high-density farming [7]. According to the literature, aerosols are a critical medium for the transmission of airborne diseases [8]. Aerosols composed of biological material are usually referred to as bioaerosols, which encompass a wide range of microorganisms and biological components, such as bacteria, fungi, viruses, spores, metabolites, and antibiotic resistance genes [9]. Infected animals generate virus-laden aerosols through activities like breathing, sneezing and coughing. Consequently, airborne transmission of pathogens occurs both directly through the inhalation of aerosols and indirectly through contact with contaminated surfaces where particles have settled [10].

Previous studies have conclusively demonstrated that various microorganisms can be found on (or attached to) aerosols of different particle sizes. Studies have shown that the majority of airborne bacteria in swine houses exhibit a size distribution with diameters greater than 3.3 μm [11,12,13]. Meanwhile, culturable bacterial counts tend to increase with particle size, indicating that these bacteria may not exist as individual particles but are likely bound to larger particles in the air [11,14,15]. Comparatively, culturable fungal counts peaked in the size range of approximately 2–5 μm, suggesting that airborne fungi may primarily exist as individual spores or particles [14,16]. Furthermore, viruses like Influenza A Virus (IAV), Porcine Reproductive and Respiratory Syndrome Virus (PRRSV), Porcine Epidemic Diarrhea Virus (PEDV), and Highly Pathogenic Avian Influenza Virus (HPAIV) were frequently detected in particles larger than 3.3 μm [17,18]. Previous studies have also detected the presence of airborne antibiotic-resistant bacteria (ARB), including methicillin-resistant Staphylococcus aureus (MRSA), in swine barns and found their geometric mean diameter to be around 7.2 μm, indicating that the majority of MRSA might be attached to larger particles [15]. Therefore, as different pathogenic microorganisms may adhere to aerosols of varying particle sizes, this could lead to differences in the transmission patterns and pathogenic mechanisms of various airborne diseases both outside of the animal’s body (in livestock housing and farms) and within the body (throughout the respiratory tract).

The current literature includes research on the pathogenesis of relevant airborne disease exploring the interaction mechanisms between viruses (such as ASFV, NDV, and IBV) and bacteria with specific host proteins (e.g., NP protein and eIF4E) and signaling pathways (e.g., cGAS/STING and PI3K/Akt/mTOR) [19,20,21,22,23]. These studies have elucidated key molecular mechanisms underlying viral replication, transcription, translation, and host immune responses, providing critical theoretical insights into the pathogenesis of airborne disease. Nevertheless, a fundamental and significant question in the study of airborne diseases remains poorly understood: the dynamics of virus-laden aerosols within the respiratory tract after inhalation by animals, as well as the deposition pattern across different regions. This issue is challenging to investigate through anatomical methods or in vivo experiments [24], and only a limited number of studies are available in the literature. For example, Sood et al. [25] demonstrated effective pulmonary aerosol delivery in neonatal pigs undergoing high-frequency oscillatory ventilation after Gadopentetate Dimeglumine (Gd-DTPA) aerosol administration. Asgharian et al. [26] proposed a mathematical model to predict the deposition of particles in the respiratory tract of pigs. Awadalla et al. [27] investigated the early airway structural changes in cystic fibrosis pigs using computational fluid dynamics (CFD) modeling. With the advancement and maturation of CFD technology, computer simulations offer a robust and versatile 3D approach, enabling researchers to better understand the dynamics of aerosols in the airways.

Furthermore, from a different yet interconnected perspective, understanding the dispersion and deposition patterns of aerosols within the respiratory tract of animals can also provide foundational scientific insights for the development of aerosolized vaccines. While needle-free administration is not novel in the livestock farming industry, exemplified by the use of spray vaccination against swine Atrophic Rhinitis (AR) [28], Newcastle Disease Virus (NDV) [29], and Infectious Bronchitis Virus (IBV) [30], research on the regional drug deposition of aerosols within the respiratory tract post-inhalation remains limited. As noted by Calderon-Nieva et al. [31], current spray vaccination methods are somehow not specifically targeted at inhalation but rather appear to induce immunity through the ocular, oral, and nasal mucosae. Thus, drawing on the well-established principles of mucosal drug administration via the pulmonary route in humans [32], a clear understanding of aerosol dynamics inside of the respiratory tract of food-producing animals would pave the way for the development of new and efficient vaccine administration via the nasal or pulmonary route in the near future.

Therefore, to bridge the gap in understanding aerosol dynamic transmission patterns and deposition distribution in the respiratory tract of animals under various respiratory conditions and to provide a more comprehensive understanding of the respiratory physiology, computed tomography (CT) scanning, 3D printing, and CFD technologies were employed in this study to develop, validate, and simulate a respiratory model of a pig. The overall objectives are as follows:

- Develop a 1:1 three-dimensional CFD model of a pig’s respiratory tract using CT scanning data.
- Validate the computational model using a 3D-printed physical model.
- Simulate the aerosol dynamics in the respiratory tract under various conditions by using the Discrete Phase Model (DPM). Qualitatively and quantitively examine the aerosol deposition behaviors.
- Reveal the penetration characteristics of aerosols with different particle sizes in the respiratory tract and the deposition distribution across various regions.
- Contribute to fundamental scientific data for the development of aerosolized vaccines by targeting distinct regions of the respiratory tract with different particle sizes.

## 2. Materials and Methods

### 2.1. Model Development and Simulation Setup

#### 2.1.1. Reconstruction of the Respiratory Tract’s Geometry

**Figure 1:**
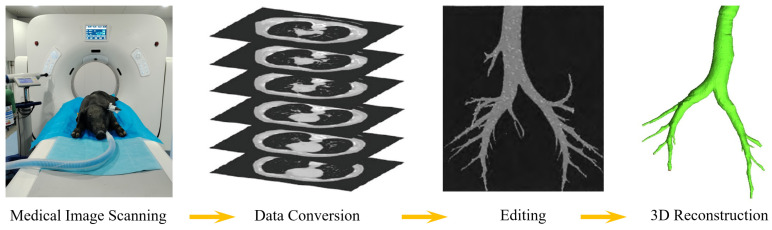
Schematic drawing of the respiratory tract geometry reconstruction process.

The geometry of the respiratory tract was reconstructed based on a computed tomography (CT) data set obtained from a healthy, ~38.5 kg male piglet. This data set comprised the nasal cavity, oropharynx, larynx, trachea, and tracheobronchial tree extending down to the fourth generation. Due to the piglet being fully anesthetized and undergoing mechanical ventilation through the trachea inserted via the mouth, the oral cavity was not reconstructed. The CT scan from the head to the chest of the animal was performed using a Quantum 752 128-Slice scanner (Kuan Teng, Beijing, China) with the settings of 500 mm field of view, 70 kV, and 50 mA. The resulting CT scan files comprised 778 contiguous images, each with a thickness of 0.5 mm, which were recorded in a Digital Imaging and Communications in Medicine (DICOM) format. Subsequently, the CT scan images were exported to image processing software (Mimics 2024) for editing, smoothing, and reconstruction. The mucus lining at the interface of the airway wall was identified through the analysis of pixel color variations within the DICOM images. Finally, the constructed geometry was converted into the Standard Tessellation Language (STL) format for the subsequent mesh generation process and 3D printing. A flow chart is provided in Figure 1 to schematically illustrate the reconstruction process.

#### 2.1.2. Mesh Generation and Regional Division

**Figure 2:**
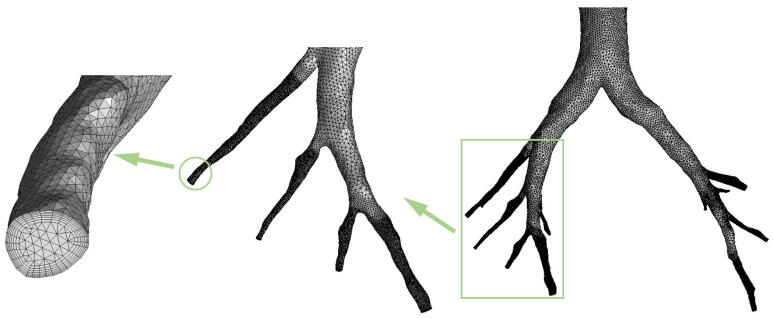
Computational mesh showing refined mesh near the airway wall.

The computational mesh of the respiratory model was generated using ANSYS ICEM 2020 software. Initially, the mesh structure was generated utilizing the octree methodology to accommodate an appropriate surface mesh. Subsequently, a method based on Delaunay triangulation was employed to produce a high-quality volume mesh. Finally, seven layers of prism mesh near the airway walls were applied to properly capture the turbulent boundary layer profile, as demonstrated in Figure 2. Special attention was paid to the height of the first layer of the prism mesh in order to ensure $y^{+}<1$ across the entire computational domain, as recommended when using the SST k−ω turbulent model.

**Figure 3:**
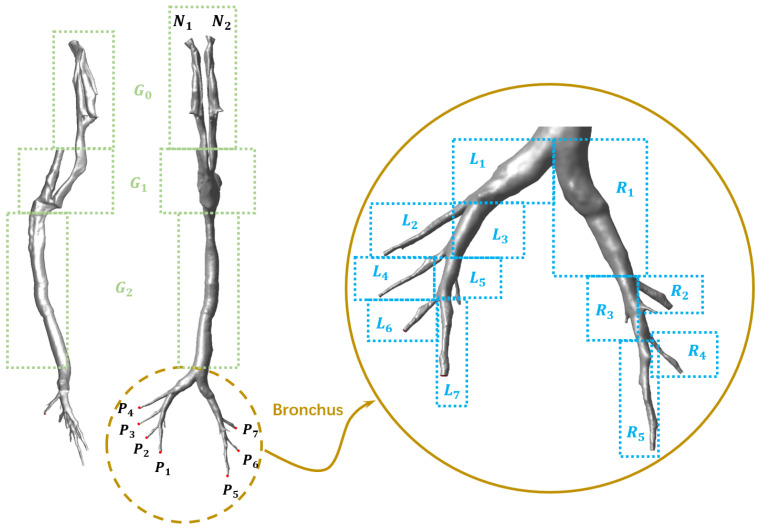
Side view, top view, and regional division of the respiratory tract.

Furthermore, in order to qualitatively and quantitatively analyze the deposition behaviors of particles with various diameters in the respiratory tract, the full geometry was divided into several segments (see Figure 3): the nasal cavity ($G_{0}$), the oropharynx and the larynx ($G_{1}$), the trachea ($G_{2}$), the main bronchus ($L_{1}\;and\;R_{1}$), the bronchial airways in the left main bronchus ($L_{2}\;\sim \;L_{7}$), and the bronchial airways in the right main bronchus ($R_{2}\;\sim \;R_{5}$). Moreover, the two nasal inlets were designated as $N_{1}$ and $N_{2}$, while the bronchial outlets were labeled $P_{1}$ through $P_{7}$, as illustrated in Figure 3.

#### 2.1.3. Particle Transport Model

The Discrete Phase Model (DPM) was applied in this study with the definition of particle initial position, velocity, diameter, temperature, etc. One-way coupling between the fluid and particles within the flow field was assumed, while interactions among particles were disregarded due to the sufficient dilution of the particulate flow, as suggested by previous studies [33]. The motion trajectory of particles was traced using the Lagrangian approach through the mean velocity field, while the turbulence effect was modeled by employing the discrete random walk (DRW) model, which incorporated a random eddy lifetime. By equating the inertia with external forces, the motion of particles was obtained as [34]

(1) $$\frac{d\vec{u_{p}}}{dt}=F_{d}\left(\vec{u}-\vec{u_{p}}\right)+\frac{\vec{g}(\rho_{b}-\rho )}{\rho_{b}}+\vec{F}$$

where $\vec{u}$ was the air velocity, $\vec{u_{p}}$ was the bioaerosol velocity, $\vec{g}$ was the gravitational acceleration, ρ was the air density, $\rho_{b}$ was the density of the bioaerosol, $F_{d}$ was the drag force, and $\vec{F}$ represented additional forces, including the Saffman lift force, the thermophoretic force, the Brownian force, and the pressure gradient force considered. Particles of specified sizes were introduced in a uniform distribution at the inlet boundary (the nose) of the computational domain and subsequently traced through the geometry. Their ultimate fate was categorized into one of three outcomes: (1) deposition on a surface via collision, (2) exit from the domain through one of the outlets or inlets, or (3) continued suspension within the flow. The outcomes of the particles were then documented and summarized in a particle history file.

The aerosols were treated as droplet-based particles in the present study, and it was assumed that the droplet aerosols consisted of volatile (92%) and non-volatile mass (8%) [35]. The volatile mass fraction consisted of water that evaporated, while a non-volatile nucleus remained. The evaporation rate of the droplet depended mainly on the water vapor concentration gradient between the droplet surface and the air environment. The droplet was controlled using the default evaporation control model of Fluent [35]. All DPM particle parameters are summarized in Table 1.

#### 2.1.4. Boundary Condition

A velocity inlet was applied at the nose to simulate piglet inhalation, with the fluid direction aligned with the normal of the inlet surfaces. The airflow velocity varied over time according to the respiratory process, and it was simplified using a sinusoidal wave, as represented by Equation (2):

(2) $$V=\frac{Q}{A}\times \sin\;(\frac{2\pi }{T}\times t)$$

where Q was the tidal volume in L/min, A was the cross area of the nose inlet in m^2^, T was the duration of one complete respiratory cycle in seconds, s, and t was the simulation time. According to the study performed by Barbosa Pereira et al. [36], the respiratory rate of piglets weighting about 37 kg was about 30 breaths/min (T ≈2s). Meanwhile, the tidal volume, Q, in this study was determined to be Q=8.5 L/min (rest state) and Q=17.0 L/min (light activity state), referencing the study performed by Su et al. [37]. Finally, the cross area of the nose inlets, A, was calculated using reconstruction software to be approximately $A\;\approx 1.41\times 10^{-4}\;m^{2}$. The bronchi outlets of $P_{1}\;\;\sim {\;P}_{7}$, as shown in Figure 3, were set as pressure outlet boundary conditions. The whole respiratory tract wall was regarded as a no-slip surface, maintained at a constant temperature of T = 39.0 °C [38], and with a species mass fraction of H_2_O of approximately 0.045, ensuring that the humidity of the air entering the lungs was nearly saturated.

Regarding the DPM boundary settings, as the airway walls were lined with sticky mucus [39], a trap condition was applied to the entire airway wall to simulate particle deposition. The escape condition was implemented at the inlets and outlets to allow particles to pass through without being reflected.

#### 2.1.5. CFD Setup

The airflow in the respiratory tract was assumed to be unsteady, incompressible, and isothermal, and it was solved using ANSYS Fluent 2020 software. The Reynolds-averaged Navier–Stokes (RANS) equations were solved using the SIMPLEC algorithm for coupling the pressure and velocity. For accuracy, the second-order upwind scheme was utilized to discretize the transport equations. Drawing upon similar research found in the literature (e.g., [40,41]), the shear stress transport (SST) k−ω turbulent model, which used the enhanced wall treatment as the default and exhibits greater accuracy in predicting the details of the wall boundary layer characteristics, was selected for the turbulence simulation. The transient time step was set to be 0.1 s, allowing convergence for all variables to be <10^−5^ between two consecutive iterations within a reasonable research timeframe. Each simulation was conducted with the same processes for comparative study, and the detailed sequences can be summarized as follows. Firstly, a converged transient solution was established (where the velocity fluctuations between adjacent cycles are nearly identical). Secondly, the DPM was activated and particles were released during the inspiratory phase (which constitutes half of a complete respiratory cycle). Finally, the release of particles was ceased, and the simulation of the expiratory phase was completed (the remaining half of a complete respiratory cycle). All simulations were repeated three times to determine the average aerosol behaviors, including deposition and escape in different regions.

#### 2.1.6. Deposition and Escape Efficiency

The regional deposition fraction of aerosols within the respiratory tract, which is usually affected by the inhalation flow rate, particle size (diameter), and anatomical complexities of the airway, provides valuable insights into the deposition patterns of inhaled particles. In this study, the deposition fraction (DF) and escape fraction (EF) were used to quantitively examine the deposition and escape characteristics of inhaled aerosols within the airway model. The DF and EF in region i are expressed as follows:

$${DF}_{i}\left(\%\right)=\frac{Number\;of\;deposited\;partilces\;in\;region\;i}{Number\;of\;injected\;particles}\times 100$$

$${EF}_{i}\left(\%\right)=\frac{Number\;of\;escaped\;partilces\;from\;outlet\;i}{Number\;of\;injected\;particles}\times 100$$

### 2.2. Grid Independency Study and Model Validation

#### 2.2.1. Grid Independency Test

**Figure 4:**
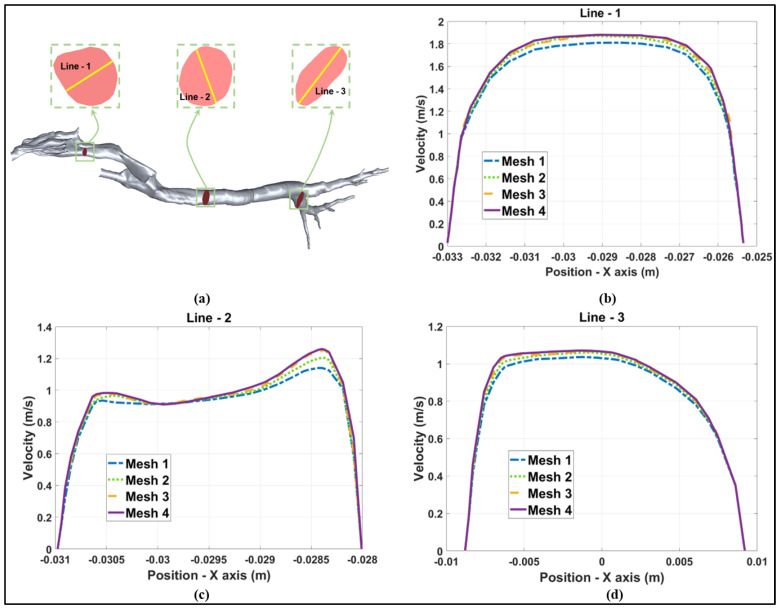
Grid independency tests at a flow rate of 8.5 L/min. (**a**) Positions of three different cross-sections around the larynx, the trachea, and the bronchus and the definition of lines. (**b**) Velocity profiles at Line-1. (**c**) Velocity profiles at Line-2. (**d**) Velocity profiles at Line-3.

Because the respiratory tract model exhibits an intricate geometry encompassing numerous anatomical complexities, a sufficiently fine mesh is indispensable for accurately capturing the geometry and yielding realistic simulation results. Therefore, a gird independency test was performed considering four meshes with varying element counts: approximately 2,220,000 (mesh 1, coarse), 2,850,000 (mesh 2, medium-coarse), 3,430,000 (mesh 3, medium-fine) and 3,810,000 (mesh 4, fine). The axial flow velocities at three cross-sections, namely, around the larynx, the trachea, and the bronchus, were plotted with the objective of examining the influence of mesh resolutions on the velocity profiles, as shown in Figure 4a. As it can be clearly seen in Figure 4b–d, by increasing the elements from 3,430,000 (mesh 3) to 3,810,000 (mesh 4), there is no significant discrepancy between the two meshes, with a maximum velocity difference of only 1.82%. Thus, considering the computing efficiency, mesh 3 was used for all of the following simulations.

#### 2.2.2. Simulation Model Validation

**Figure 5:**
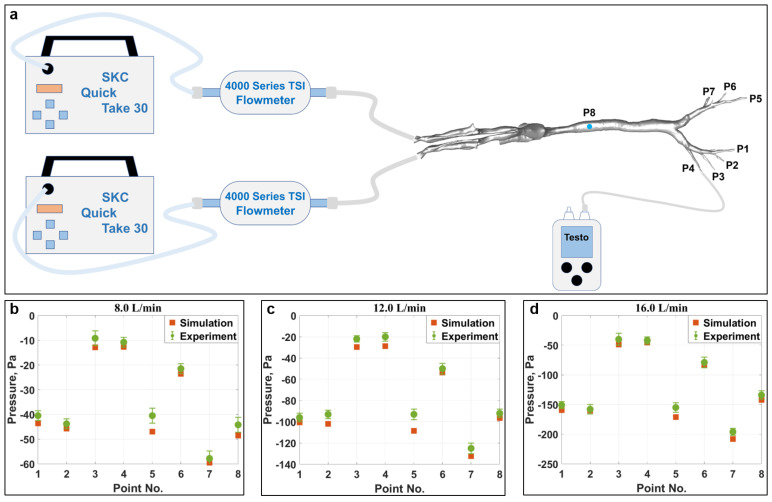
(**a**) Schematic drawing of the experimental setup for measuring pressure at points P1 to P8. Comparison of the measured and simulated pressure values at flow rates of (**b**) 8.0 L/min, (**c**) 12.0 L/min, and (**d**) 16.0 L/min.

In order to validate the developed 3D simulation model, a 1:1 high-resolution physical model of the respiratory tract was manufactured using 3D printing technology, as illustrated in Table 2. The Acrylonitrile Butadiene Styrene (ABS) material was selected due to its affordable price and satisfactory strength. Pumps equipped with flow meters were used to provide an accurate airflow rate, and a pressure meter was utilized to measure the pressure values at various points, as indicated in Figure 5a. Specifically, points P1 ~ P7 were located at the end of the bronchus (outlets), and an additional location (Point 8), created by drilling at the trachea, was chosen for validation. Detailed information about the experimental equipment is summarized in Table 2. Three airflow rates (8.0 L/min, 12.0 L/min, and 16.0 L/min), which encompassed the range of respiratory volumes intended for subsequent simulations, were examined, and the results are shown in Figure 5b–d. Generally speaking, the overall simulated values correspond well with the measured pressure data. It is hypothesized that the slightly higher pressures (low negative pressure values) measured during experiments are due to the relatively high surface roughness of the unpolished internal surface of the physical model (lower velocity). In contrast, the surface roughness effect is not accounted for during numerical simulations. Except for Point 5, the maximum relative difference for all other points under three examined airflow rates is calculated to be about 9.8%, indicating good agreement [42]. Regarding Point 5, situated at the farthest end of the bronchus, the comparatively small aperture size may lead to imperfections in the replication process during 3D printing, thereby contributing to the observed discrepancies, which are relatively large but still acceptable. The corresponding root mean square error (RMSE) for airflow rates of 8.0 L/min, 12.0 L/min, and 16.0 L/min is limited to be only 2.8 Pa, 7.4 Pa, and 8.9 Pa, respectively. Taking the complexity of both the turbulence flow and model geometry into consideration, the simulated outcomes are deemed acceptable, and the model is capable of providing reasonable predictions [43,44,45], thereby confirming the validity of the developed numerical model.

## 3. Results and Discussion

**Figure 6:**
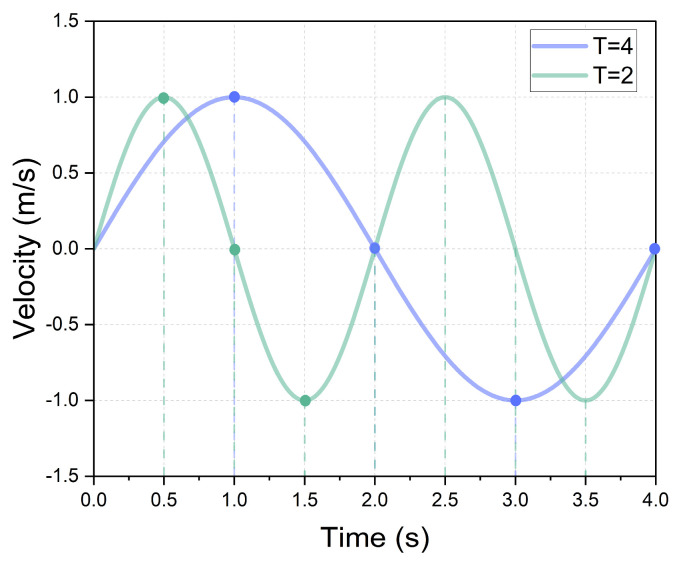
Simplified sinusoidal respiratory process with Q = 8.5 L/min for respiratory cycles of T = 2 s and T = 4 s.

Several parameters, such as airway geometry and the inhalation flow rate, can significantly impact the flow structure and, consequently, subsequent aerosol deposition. Moreover, the aerosol diameter also plays a crucial role in its deposition mechanisms, including impaction, sedimentation, and diffusion, as elaborated in the existing literature [46]. Therefore, in this study, we provided comprehensive analyses under two flow conditions: a rest state with Q = 8.5 L/min and a light activity state with Q = 17.0 L/min. In addition to the normal duration of one complete respiratory cycle of T = 2 s, we also examined the effects of a different respiratory cycle (T = 4 s) on the flow characteristics and aerosol depositions, as exemplified in Figure 6. All studies were conducted for six aerosol diameters (0.1 μm, 1.0 μm, 2.5 μm, 5.0 μm, 10.0 μm, and 20.0 μm), which fall within the typical size ranges found in livestock houses [9,47] and are also within the common size ranges used in drug delivery and aerosolized vaccines [33,48].

### 3.1. Flow Structure and Characteristics

#### 3.1.1. Streamlines of Airflow in Typical Regions of the Respiratory Tract

**Figure 7:**
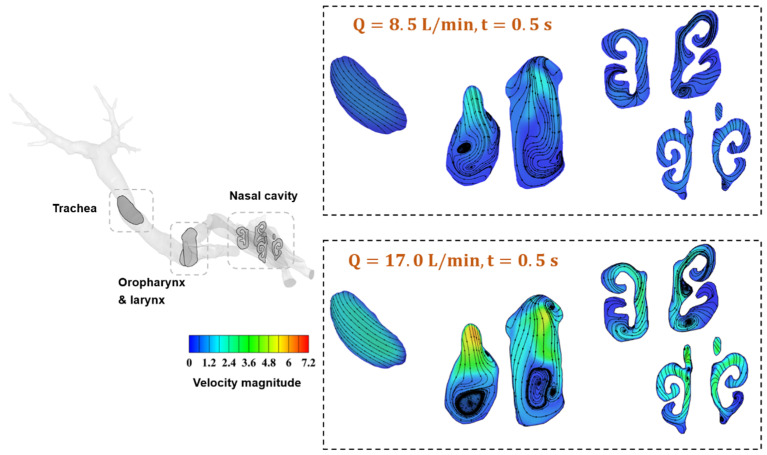
Airflow streamlines at key regions of the nasal cavity, the oropharynx, the larynx, and the trachea at t = 0.5 s for the respiratory cycle of T = 2 s.

Figure 7 illustrates the streamlines and velocity magnitude at cross-sections of typical regions in the respiratory tract. At the flow rate of Q = 8.5 L/min, vortices can be easily identified in the nasal cavity due to its unique and complex anatomy. Moreover, the oropharynx and larynx form a nearly 90° bend, producing a turbulent flow field that induces airflow reversal in the extrathoracic region. Because most aerosol deposition in the extrathoracic region is attributed to the turbulent flow field and the inertia of large micron-sized particles leads to impaction at the bend [33], this structure acts as a filter for large inhaled particles. Furthermore, by increasing the flow rate to Q = 17.0 L/min, more vortices can be clearly observed due to the enhanced turbulent level, especially in the oropharynx and larynx regions. An interesting phenomenon worth noting is that the airway encounters a constriction at the larynx, which accelerates the flow and results in a jet known as the laryngeal jet [49]. Consequently, the velocity magnitude around the larynx is higher than that in other regions, as seen in Figure 7. Previous studies in humans have demonstrated that the laryngeal jet enhances the deposition of inhaled particles due to increased turbulence, a finding that is also confirmed in the current study and will be detailed in the following sections. Finally, turbulence intensity in the trachea and bronchial airways gradually decreases towards more distal generations, where sedimentation becomes more effective [50]. Similar results and conclusions are obtained for the respiratory cycle of T = 4 s, and figures are provided in the Supplementary Material (Figure S1).

#### 3.1.2. Dynamic Velocity and Pressure Pattern in the Airway

**Figure 8:**
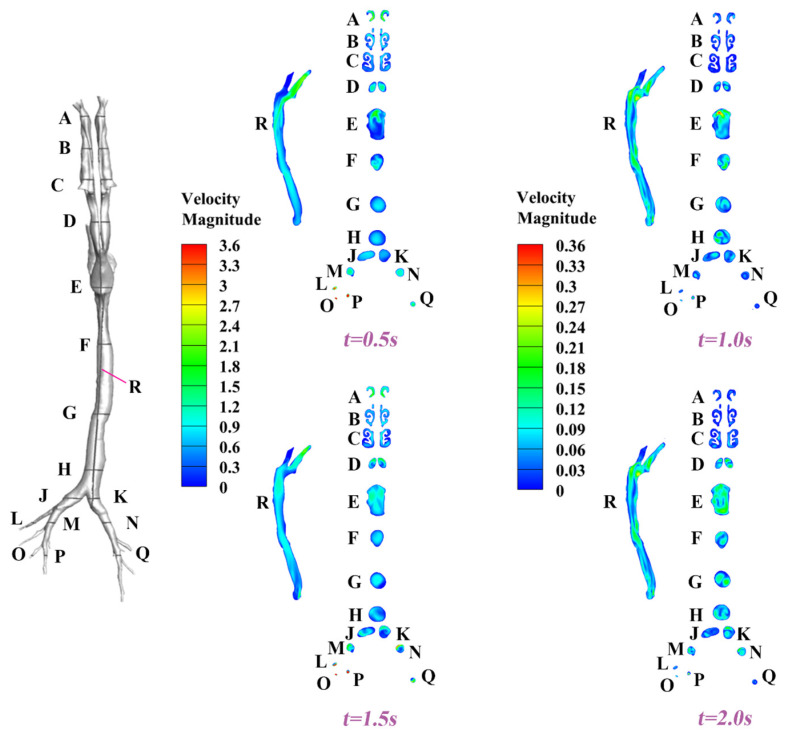
Velocity contours for the dynamic respiratory cycles at t = 0.5 s, 1.0 s, 1.5 s, and 2.0 s under Q = 8.5 L/min and T = 2 s.

**Figure 9:**
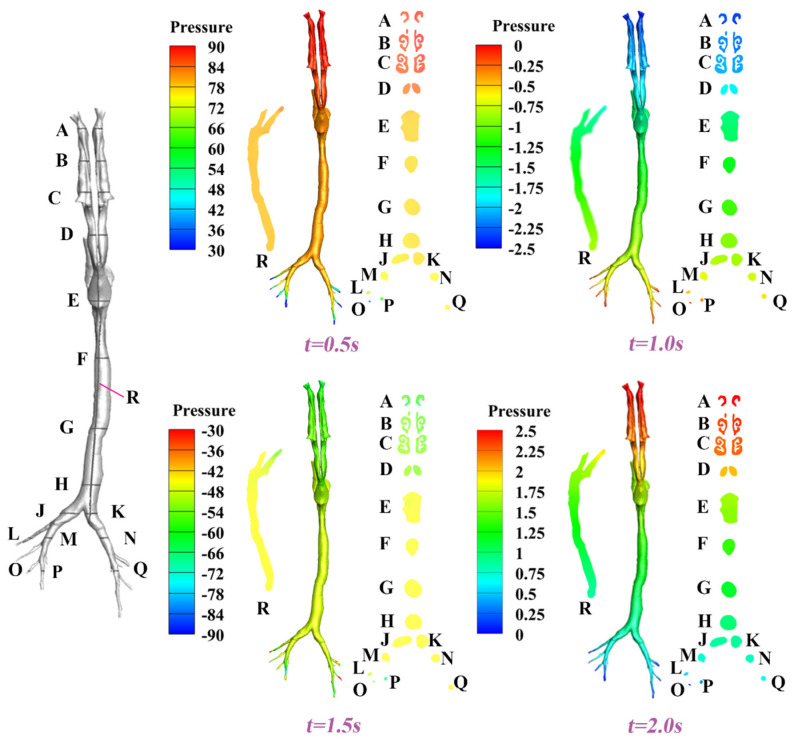
Pressure contours for the dynamic respiratory cycles at t = 0.5 s, 1.0 s, 1.5 s, and 2.0 s under Q = 8.5 L/min and T = 2 s.

In this section, we present spatiotemporally resolved velocity and pressure contours characterizing the complete respiratory cycle. To facilitate three-dimensional analysis, several transverse cross-sections (surfaces A–Q) and a longitudinal cross-section (surface R) were strategically positioned along the airway to provide a comprehensive view of the flow characteristics. The corresponding results for velocity and pressure under Q = 8.5 L/min and T = 2 s are depicted in Figure 8 and Figure 9, respectively. In general, owing to the inherent anatomical asymmetry of the nasal structure, bilateral asymmetry in both velocity profiles and pressure distributions is observed. Progressive airflow deceleration accompanied by pressure recovery is observed as the cross-sectional area of the nasal cavity gradually expands. The converging airstreams from bilateral nasal passages form a characteristic laryngeal jet in the oropharyngeal region. At the peak inhalation point (t = 0.5 s, referring to Figure 6), maximum velocities of around 3.6 m/s and minimum pressures of around 30 Pa are detected at the terminal bronchioles, where the minimization of the cross-sectional area amplifies flow acceleration. Time-resolved analysis revealed non-uniform airway filling patterns, demonstrating that the airway geometry induces complex secondary flow structures and temporal flow instabilities, as exemplified by surfaces D, E, and F at t = 0.5 s, 1.0 s, 1.5 s, and 2.0 s.

Furthermore, as evidenced in Figure 9, distinct pressure distribution asymmetries were predicted between the inspiratory phase (for example, the peak inspiratory point at t = 0.5 s) and the expiratory phase (for example, the peak expiratory point at t = 1.5 s). This quantitatively confirms the absence of bidirectional flow symmetry resulting from the aforementioned unsteady flow characteristics, which is consistent with previous studies in humans [51]. Meanwhile, such phase-dependent flow characteristics suggest that airway geometry dominates the unsteady energy transfer between kinetic and pressure components, which may critically influence aerosol transport behaviors and mucociliary clearance mechanisms. Additionally, when comparing results from a different respiratory cycle of T = 4 s (Figures S2 and S3 in the Supplementary Material), it is found that at characteristic moments (such as the peak inspiratory and expiratory points), the ranges of velocity magnitudes and pressure patterns are similar, with only limited differences. This suggests that differences in respiratory cycles are primarily reflected in the timing of pressure changes at these characteristic moments. Conversely, it is the respiratory rate that is the key factor influencing the distribution of pressure in the respiratory tract. The above findings provide a scientific basis for investigating the level of wall injury in the respiratory tract and developing corresponding therapeutic measures for pre- and post-surgery, if needed [52].

### 3.2. Visualization of Aerosol Dynamic Deposition

**Figure 10:**
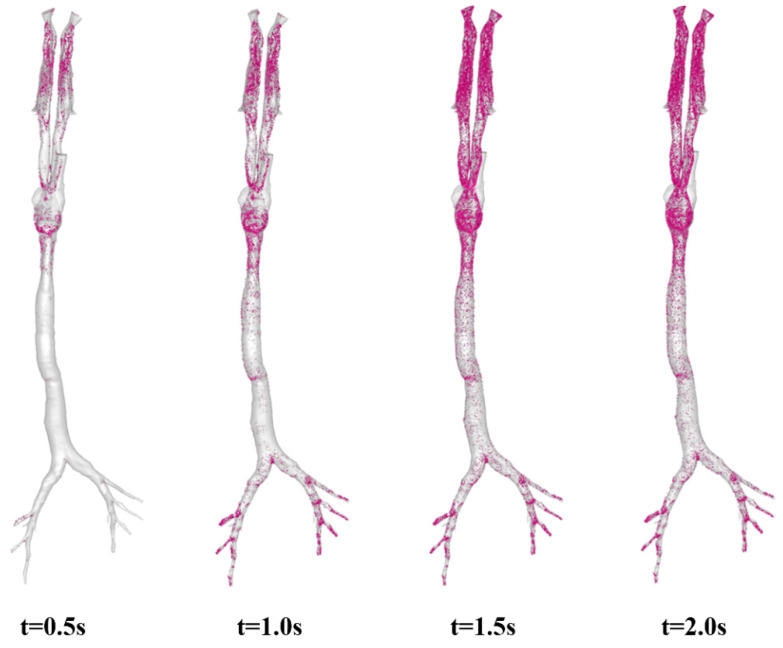
Aerosol deposition at characteristic moments: t = 0.5 s (peak inhalation point), t = 1.0 s (end of inspiratory phase), t = 1.5 s (peak expiratory point), and t = 2.0 s (end of a respiratory cycle). Q = 8.5 L/min, T = 2 s, and aerosol dimeter D = 2.5 μm.

In this part, visualized images are provided to offer a qualitative analysis of aerosol dynamic deposition in the airways. Figure 10 shows the aerosol deposition at characteristic moments under the respiratory cycle of T = 2 s. In total, 69.4% of the total number of deposited aerosols occurs during the time interval t = 0 ~ 0.5 s. Due to the long nasal cavity and complex anatomical structure of pigs, a significant amount of aerosol deposition is observed in this region. Additionally, the presence of the laryngeal jet results in substantial aerosol deposition around the oropharynx and the larynx. For micron-sized particles, such as D = 2.5 μm, inertial impaction is the predominant deposition mechanism in the above-mentioned regions [53]. However, due to the diffusion effect, these small particles can also move in the crossflow direction and reach the inner airway, even with an almost vertical bend in the laryngeal region [54]. Ultimately, a small portion of aerosols reached the lower respiratory tract and was deposited at the distal bronchial generations by t = 0.5 s. By the end of the inspiratory phase (t = 1.0 s), the deposited aerosols account for about 89.3% of the total deposition, with the remaining 10.7% occurring during the entire expiratory phase from t = 1.0 s to t = 2.0 s. Several major deposition sites can be clearly identified: the nasal cavity, the oropharynx, the larynx, and the distal bronchi. Due to the low level of turbulence, only a limited number of aerosols are deposited in the trachea region. Because the majority of aerosol deposition takes place during the inspiratory phase, almost identical images were observed at the two expiratory time points of t = 1.5 s and t = 2.0 s, as shown in Figure 10.

**Figure 11:**
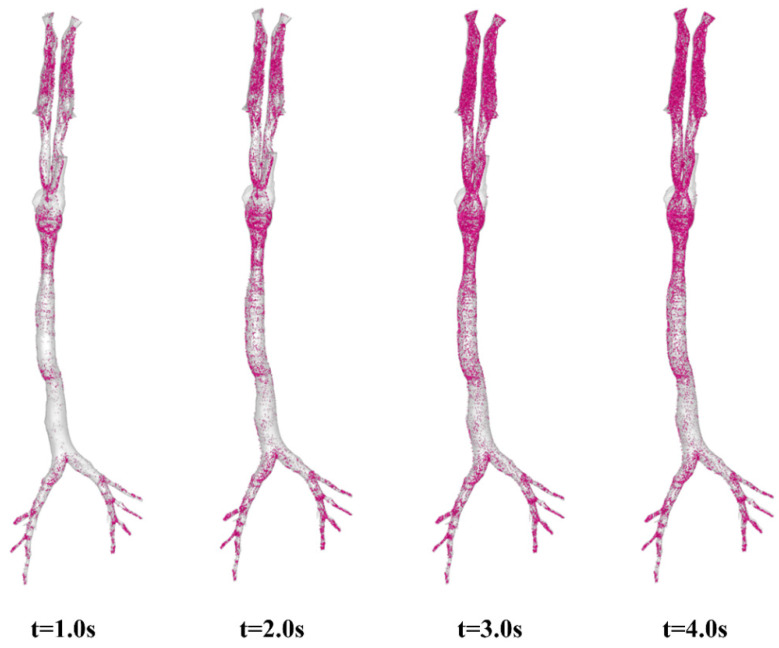
Aerosol deposition at characteristic moments: t = 1.0 s (peak inhalation point), t = 2.0 s (end of inspiratory phase), t = 3.0 s (peak expiratory point), and t = 4.0 s (end of a respiratory cycle). Q = 8.5 L/min, T = 4 s, and aerosol dimeter D = 2.5 μm.

When the respiratory cycle is increased from T = 2 s to T = 4 s, as illustrated in Figure 11, a different story is observed. The corresponding ratios of deposited aerosols at characteristic moments to the total deposition are 84.6% (t = 1.0 s, peak inhalation point), 97.9% (t = 2.0 s end of inspiratory phase), 99.9% (t = 3.0 s, peak expiratory point) and 100% (end of the respiratory cycle), respectively. Because the majority of the deposited aerosols are captured by the airway walls before t = 1.0 s, the four images in Figure 11 appear quite similar. It is believed that a longer respiratory cycle allows aerosols inhaled through the noses to have more time to migrate and be captured by the airway walls during the inspiratory phase (t = 0 ~ 2.0 s), as well as a greater chance of depositing in the lower respiratory tract or escaping into the lungs. Furthermore, we also investigated the impact of different inhalation rates (Q = 17.0 L/min) on aerosol behaviors in the airways (see Figures S4 and S5 in the Supplementary Material). The results demonstrated conclusively that the higher the inhalation rate and the longer the respiratory cycle, the more likely the particles are to reach the lower respiratory tract and deposit in a shorter time, yet the primary deposition regions remained unchanged.

### 3.3. Regional Escape and Deposition Fraction

**Figure 12:**
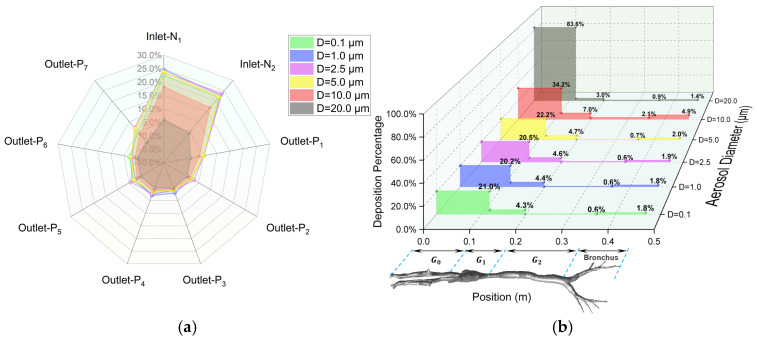
Regional (**a**) escape fraction (EF) and (**b**) deposition fraction (DF) under Q = 8.5 L/min, T = 2 s. The naming of the inlets and outlets refers to Figure 3.

The regional deposition and escape efficiency (as detailed in Section 2.1.6) and the effects of aerosol diameter on dynamic characteristics are quantitatively examined in this section. Firstly, as demonstrated in Figure 12, numerical predictions demonstrate negligible size-dependent variation for aerosols with D ≤ 5.0 μm. Upon completion of a full respiratory cycle, 47.3% of inhaled aerosols are exhaled through the nasal cavity, while 21.1% of the total released aerosols penetrate into the lungs through the seven terminal bronchioles of the lower airways (outlets P1 ~ P7). Figure 12a, in the form of a radar chart, provides a detailed illustration of the escape ratios at each inlet and outlet. Specifically, outlets P1, P4, P5, and P7 are identified as the primary pathways for aerosols to enter the lungs. Total airway wall deposition accounts for approximately 24% ~ 27% of the inhaled aerosols, with a limited number of particles remaining airborne at the end of the respiratory cycle. Furthermore, Figure 12b provides a 3D illustration of the detailed deposition percentage in each region of the respiratory tract wall. Within its total deposition ratio of 27.5%, the majority, approximately 20 ~ 22%, is deposited in the G0 region (the nasal cavity). The G1 region (the oropharynx and larynx) accounts for about 4.5%, the lower respiratory tract (bronchus) for about 1.9%, and the G2 region (the trachea) for the least, at about 0.6%.

Secondly, the deposition behaviors of larger aerosols are significantly different from those of smaller ones. For aerosols with D = 10.0 μm, Figure 12a clearly shows a substantial reduction in the total percentage of particles escaping from the inlets and outlets at the end of a respiratory cycle (indicated by the area enclosed by the red line for D = 10 μm). Specifically, the proportion of aerosols escaping into the lungs (outlets P1 ~ P7) decreases considerably to approximately 15.8%, while the majority of aerosols (48.2%) are deposited on the airway walls. This is further corroborated by Figure 12b, which shows that for D = 10 μm, the deposition percentage in every region of the respiratory tract is higher than that for smaller particles. When the aerosol size increases to D = 20 μm, the influence of gravity causes the majority of particles entering the respiratory tract to deposit rapidly in the nasal cavity (83.6%), with only a small fraction reaching the larynx (3.0%) and the lower respiratory tract (2.3%). Consequently, virtually no aerosols escape into the lungs. Additionally, about 11.1% of the inhaled aerosols are exhaled again during the expiratory phase.

**Figure 13:**
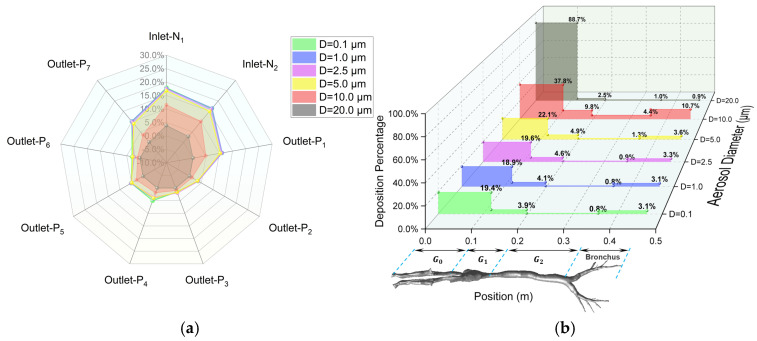
Regional (**a**) escape fraction (EF) and (**b**) deposition fraction (DF) under Q = 8.5 L/min, T = 4 s. The naming of the inlets and outlets refers to Figure 3.

Thirdly, when the respiratory cycle is increased to T = 4 s while keeping the inhalation rate at Q = 8.5 L/min (see Figure 13), it is observed that for aerosols with D ≤ 5.0 μm, the prolonged residence time enables enhanced migration and diffusion within the airways, resulting in a substantial increase in pulmonary penetration through the outlets p1 ~ p7 (37.8%), despite maintaining a comparable airway wall deposition efficiency of about 27 ~ 32% for the T = 2 s condition. In contrast, respiratory cycle variations exhibit limited influence on dynamics of large aerosols with D = 10.0 μm and 20.0 μm, as evidenced by a proportional reduction in the percentage of nasal escaping that closely matches the compensatory increase in airway wall deposition.

Fourthly, when the inhalation rate is increased to Q = 17.0 L/min while maintaining the respiratory cycle at T = 2 s (see Figure S6 in the Supplementary Material), it is found that increased inhalation rates significantly alter the dynamics of aerosols with D ≤ 5.0 μm, demonstrating an enhanced capability of pulmonary penetration (34.5%). The proportion of particles expelled through the nasal cavity is significantly reduced, with these particles instead being deposited on the respiratory tract wall, particularly in the upper respiratory tract. It is worth noting that for aerosols with D = 10 μm, increasing the inhalation rate surprisingly reduces their capability of escaping into the lungs. This may be because, for larger particles, an increase in the turbulence level reduces their probability of passing through complex structures, such as the oropharynx and the larynx, due to the effect of inertia impaction, causing them to deposit on the respiratory tract wall instead. Moreover, for even larger particles with D = 20 μm, both the inhalation rate and the respiratory cycle have minimal impact on the results, with the particles rapidly depositing in the nasal cavity.

Finally, it is worth noting that an apparent asymmetry in particle deposition is observed between the left and right bronchial airways. Detailed deposition studies in the lower respiratory tract regions (see Table 3) reveal that the deposition ratio between the left and right sides is approximately 0.83 (note: due to the mirrored nature of CT scan images and related modeling, the label “L” in Figure 6 and Table 3 corresponds to the physical right side, while “R” corresponds to the physical left side). Furthermore, monitoring the total airflow in the left and right bronchial airways over an entire respiratory cycle shows that the airflow ratio is about *Q_L_*_/*R*_ = 0.89, independent of the respiratory cycle. After reviewing the literature and analyzing the anatomical structure of the CT scans, it is believed that the reason for this uneven deposition can be attributed to the distinct geometries of the bronchial airways in the left and right lungs. As the heart is not centrally located in the thoracic cavity but slightly offset to the left (see Figure S7 of CT scan images from the present study in the Supplementary Material), it shares space with the left lung, resulting in the left lung’s bronchial airways being narrower and longer than those in the right lung (also see Figure 3). Consequently, the uneven distribution of the inhaled airflow results in higher airflow in the right airway branches, thereby leading to enhanced deposition, a finding consistent with observations in human airways [33].

### 3.4. Limitations

In this study, we systematically investigated the transient velocity fields, pressure distributions, and aerosol dynamics at multiple characteristic moments within the respiratory tract by modeling the breathing process as a sinusoidal waveform. While experimental data on authentic respiratory rhythms remained unavailable in the existing literature, the sinusoidal simplification demonstrated superior fidelity to actual physiological conditions compared to the steady-state simulation methods commonly employed in previous studies [46,55]. Although this idealized assumption might introduce some deviations in the prediction, its dynamic characteristics provided essential foundations for elucidating complex flow phenomena in respiratory systems. Moreover, the constant temperature and humidity boundary conditions applied in the present model would, to some extent, affect the behaviors of the aerosols, which could be considered variable in future studies.

Furthermore, it is important to note that the respiratory model used in this study did not account for the presence and function of airway mucus and cilia. Airway mucus and cilia played a crucial role in mucociliary clearance, a primary defense mechanism of the respiratory system, by trapping inhaled particles and maintaining the cleanliness and patency of the airways [56]. Additionally, cilia were involved in sensing the external environment and could initiate immune responses upon detecting harmful substances [57]. While the omission of mucus and cilia in our model might introduce some deviations in the simulation results, particularly by overestimating the escape and deposition of aerosols in the lower airway, it is worth mentioning that the existing literature on aerosol dynamics in the respiratory tract in humans has also overlooked the impact of mucus and cilia [51]. Future studies should consider incorporating the effects of cilia and mucus to provide a more comprehensive understanding of aerosol behavior and its implications for respiratory health.

## 4. Conclusions

To fill the knowledge gap regarding the patterns of aerosol dynamic transmission and the distribution of deposition within the respiratory systems of food-producing animals under diverse respiratory conditions, the geometry of a pig’s respiratory tract, including the nasal cavity, the oropharynx, the larynx, the trachea, and the tracheobronchial tree down to the distal generations, was reconstructed using CT scan technology. Numerical models were then developed and validated by comparing predicted results with experimental measurements obtained from a 1:1 scale physical model of the entire airway, which was produced using 3D printing techniques. CFD simulations were performed to predict the velocity contours and pressure distributions within the airway at various regions under a dynamic respiratory process modeled by a sinusoidal waveform. Moreover, the DPM was applied to understand the aerosol dynamics inside of the respiratory tract, including transmission, deposition, and escape characteristics. Different respiratory cycles and inhalation rates were examined in conjunction with various aerosol diameters, which were representative of typical size ranges found in livestock houses and commonly used in drug delivery and aerosolized vaccines, to provide comprehensive insights. Based on the results from the present study, several important conclusions could be drawn. as follows.

- Aerosol Screening Mechanism Driven by Anatomical–Fluid Coupling Effect.

The extended nasal cavity and the near 90° bend around the pharynx of pigs create a turbulent flow field that, in combination with inertial impaction, efficiently intercepts aerosols with particle sizes D ≥ 10.0 μm. In contrast, submicron particles with D ≤ 5.0 μm can penetrate the upper respiratory tract barrier through diffusion. This anatomical–fluid coupling acts as a “natural filter”, explaining the differential transmission risks of pathogen aerosols of varying sizes and providing a theoretical basis for targeted interception of key-sized pathogens.

- Particle-Size-Dependent Regulation Patterns of Respiratory Dynamics Parameters.

Under resting conditions, for aerosols with D ≤ 5.0 μm, 21.1% of inhaled aerosols underwent pulmonary deposition by the end of a complete respiratory cycle, while 27.5% was deposited on respiratory tract walls. In contrast, larger aerosols with D = 10.0 μm exhibited a marked reduction in pulmonary penetration efficiency, with only 15.8% reaching the lungs and a predominant deposition rate of 48.2% on respiratory tract surfaces. For aerosols withD = 20.0 μm, direct nasal cavity deposition accounted for 83.6% of total particles, with negligible penetration into the pulmonary region. Moreover, doubling the respiratory cycle or inhalation rate could significantly enhance the penetration ability of small-sized aerosols (D ≤ 5.0 μm) into the lower respiratory tract by 60% ~ 70%. However, for aerosols D ≥ 10.0 μm, increasing the respiratory cycle or inhalation rate led to a further reduction in their likelihood of traversing complex anatomical structures, such as the oropharynx and the larynx, due to inertial impaction, thereby causing them to quickly deposit on the walls of the upper respiratory tract instead. These results reveal a nonlinear interaction between respiratory parameters and particle size, offering dynamic regulatory approaches for optimizing vaccine aerosol particle size and respiratory intervention strategies.

- Bronchial Deposition Asymmetry Induced by Cardiac Anatomical Bias.

The leftward position of the pig’s heart results in a narrower left bronchus compared to the right side, leading to airflow distribution and deposition ratios of 0.89 and 0.83, respectively. This anatomical–fluid–deposition spatial coupling effect quantifies the impact of organ space competition on respiratory tract aerosol distribution, providing a biomechanical foundation for the asymmetric design of precision pulmonary targeting drug delivery systems.

## Figures and Tables

**Table 1:** DPM particle parameters.

	Value	Unit
Particle type	Droplet	-
Volatile mass fraction	92	%
Injection type	Surface injection	-
Particle initial velocity	0	m/s
Particle initial temperature	23	°C
Density	1000	kg/m^3^
Mass flow rate	1 × 10^−12^	kg/s
Particle diameter	0.1, 1.0, 2.5, 5.0 10.0, 20.0	μm

**Table 2:** Detailed information about the experimental equipment.

Name	Type/Manufacturer	Unit	Resolution	Image
3D-printed 1:1 respiratory tract model	Acrylonitrile Butadiene Styrene (ABS)	-	-	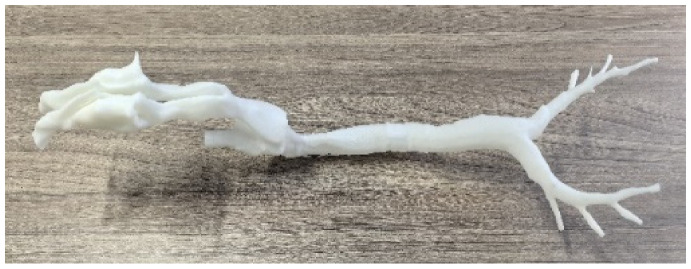
Air pump	Quick Take 30, SKC, Eighty Four, PA, USA	L/min	0.01	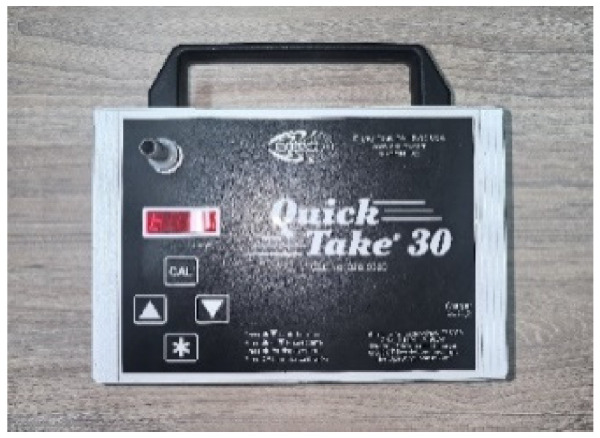
Flow meter	4000 Series, TSI, Shoreview, MN, USA	L/min	0.01	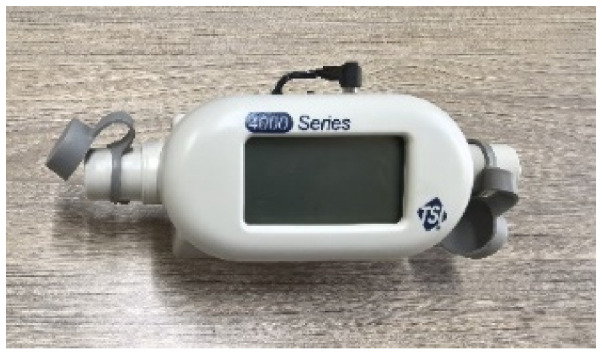
Pressure meter	Testo 510, Titisee-Neustadt, Baden-Württemberg, Germany	Pa	1.0	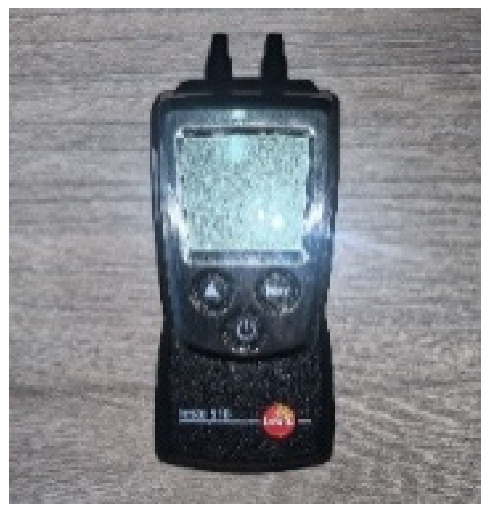

**Table 3:** Aerosol deposition percentages in the bronchial airways for Q = 8.5 L/min, T = 2 s.

Regional DF	D = 0.1 μm	D = 1.0 μm	D = 2.5 μm	D = 5.0 μm	D = 10.0 μm
L1	0.11%	0.10%	0.10%	0.09%	0.25%
L2	0.14%	0.15%	0.15%	0.17%	0.38%
L3	0.16%	0.16%	0.16%	0.18%	0.43%
L4	0.12%	0.13%	0.14%	0.15%	0.33%
L5	0.16%	0.16%	0.16%	0.17%	0.39%
L6	0.10%	0.11%	0.12%	0.13%	0.22%
L7	0.18%	0.19%	0.19%	0.22%	0.63%
R1	0.16%	0.16%	0.17%	0.17%	0.44%
R2	0.17%	0.18%	0.19%	0.22%	0.56%
R3	0.17%	0.17%	0.17%	0.19%	0.52%
R4	0.11%	0.12%	0.13%	0.15%	0.40%
R5	0.19%	0.19%	0.19%	0.18%	0.31%
L/R ratio	1.21	1.22	1.20	1.22	1.18

Note: due to the mirrored nature of CT scan images and related modeling, the label “L” in Figure 3 and Table 3 corresponds to the physical right side, while “R” corresponds to the physical left side.

## Data Availability

Data will be made available upon request.

## Supplementary Materials

The following supporting information can be downloaded at: https://www.mdpi.com/article/10.3390/ani15101396/s1, Figure S1: Airflow streamlines at key regions of the nasal cavity, oropharynx, larynx and trachea at t = 1.0 s for the respiratory cycle of T = 4 s.; Figure S2: Velocity contours for the dynamic respiratory cycles at t = 0.5 s, 1.0 s, 1.5 s & 2.0 s under Q = 8.5 L/min & T = 4 s.; Figure S3: Pressure contours for the dynamic respiratory cycles at t = 0.5 s, 1.0 s, 1.5 s & 2.0 s under Q = 8.5 L/min & T = 4 s.; Figure S4: Aerosol deposition at characteristic moments: t = 0.5 s (peak inhalation point), t = 1.0 s (end of inspiratory phase), t = 1.5 s (peak expiratory point) and t = 2.0 s (end of a respiratory cycle). Q = 17.0 L/min, T = 2 s and aerosol dimeter D = 2.5 μm.; Figure S5: Aerosol deposition at characteristic moments: t = 1.0 s (peak inhalation point), t = 2.0 s (end of inspiratory phase), t = 3.0 s (peak expiratory point) and t = 4.0 s (end of a respiratory cycle). Q = 17.0 L/min, T = 4 s and aerosol dimeter D = 2.5 μm.; Figure S6: Regional (a) escape and (b) deposition fraction under Q = 17.0 L/min, T = 2 s. The naming of the inlets and outlets refers to Figure 3; Figure S7: CT scan images of the pig showing the heart offset to the left. Note the left (L) and right (R) labels on the images.
