# Supplementary material for: Aerosol Dynamics in the Respiratory Tract of Food-Producing Animals: An Insight into Transmission Patterns and Deposition Distribution
Source: Animals (Basel). 2025 May 12;15(10):1396. doi: 10.3390/ani15101396 (PMC12108156; doi:10.3390/ani15101396)
Supplement: Supplementary file 1 [file animals-15-01396-s001.zip › animals-3614250-supplementary.pdf]

Supplementary Material

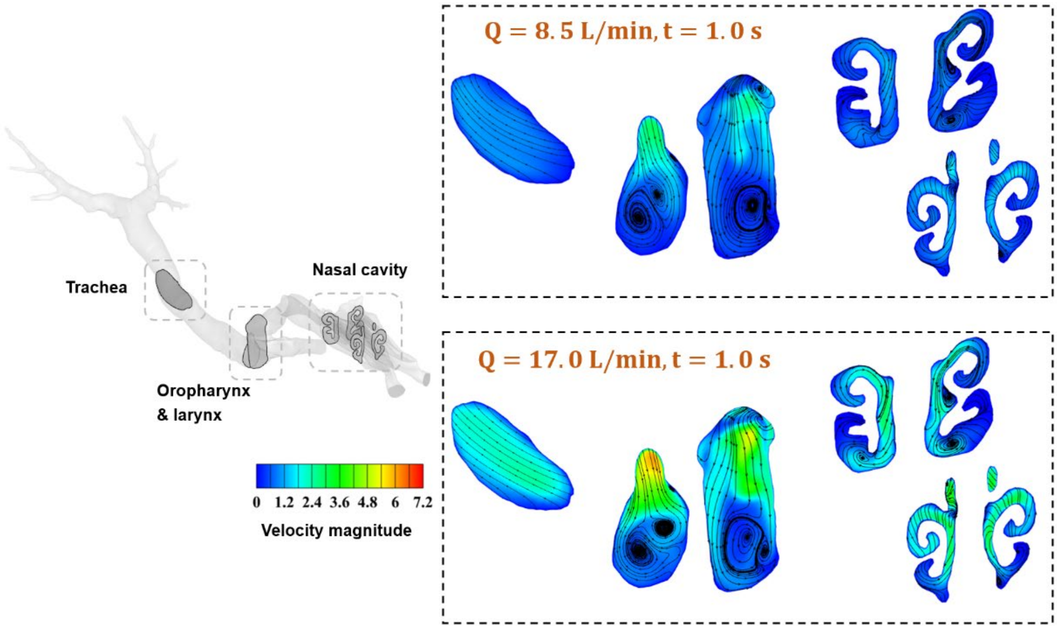

Figure S1 Airflow streamlines at key regions of the nasal cavity, oropharynx, larynx and trachea at $t = 1.0\ \mathrm{s}$ for the respiratory cycle of $T = 4\ \mathrm{s}$.

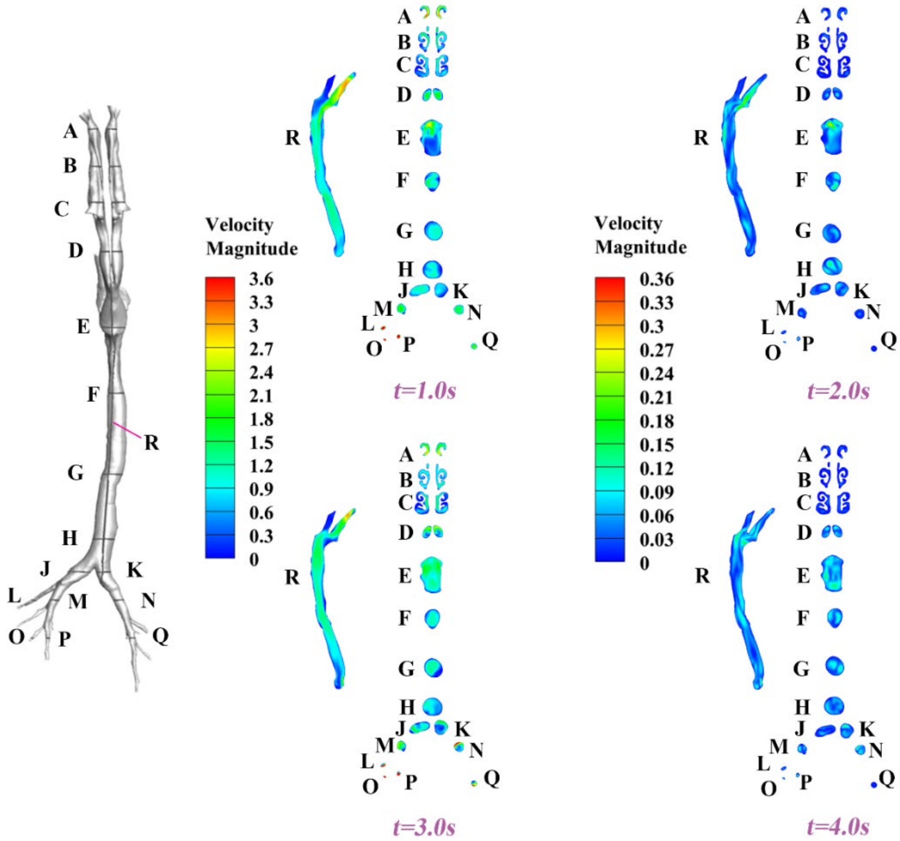

Figure S2 Velocity contours for the dynamic respiratory cycles at $t = 0.5\ \mathrm{s}, 1.0\ \mathrm{s}, 1.5\ \mathrm{s}\ \&\ 2.0\ \mathrm{s}$ under $Q = 8.5\ \mathrm{L/min}\ \&\ T = 4\ \mathrm{s}$

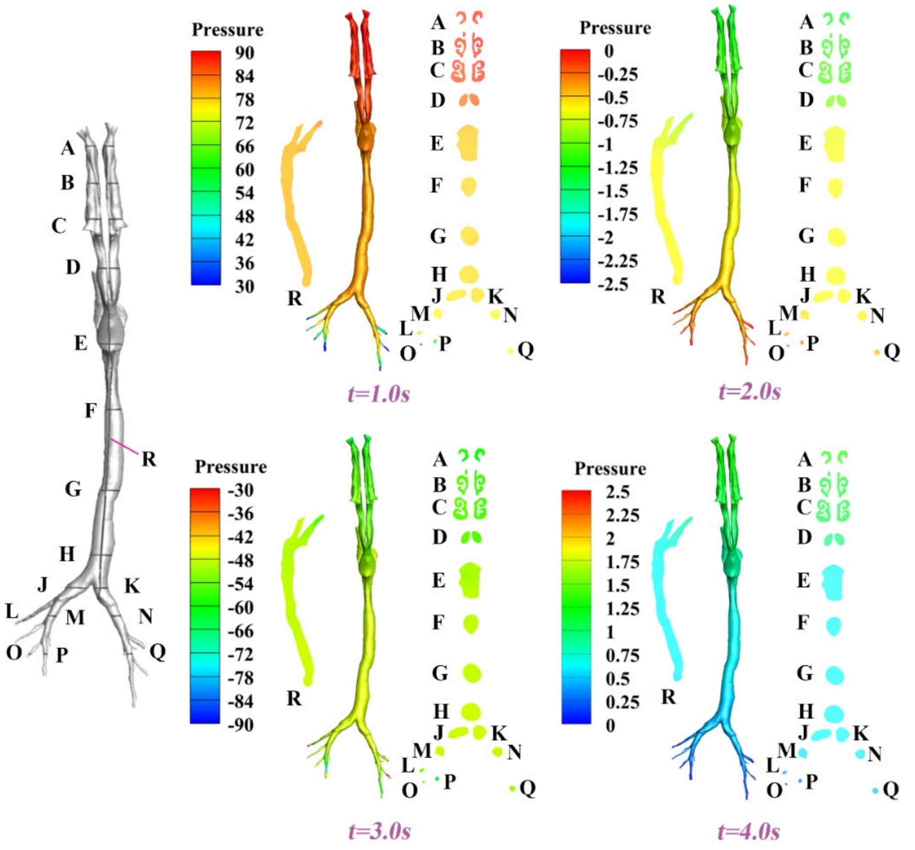

Figure S3 Pressure contours for the dynamic respiratory cycles at $t = 0.5$ s, 1.0 s, 1.5 s & 2.0 s under $Q = 8.5$ L/min & $T = 4$ s

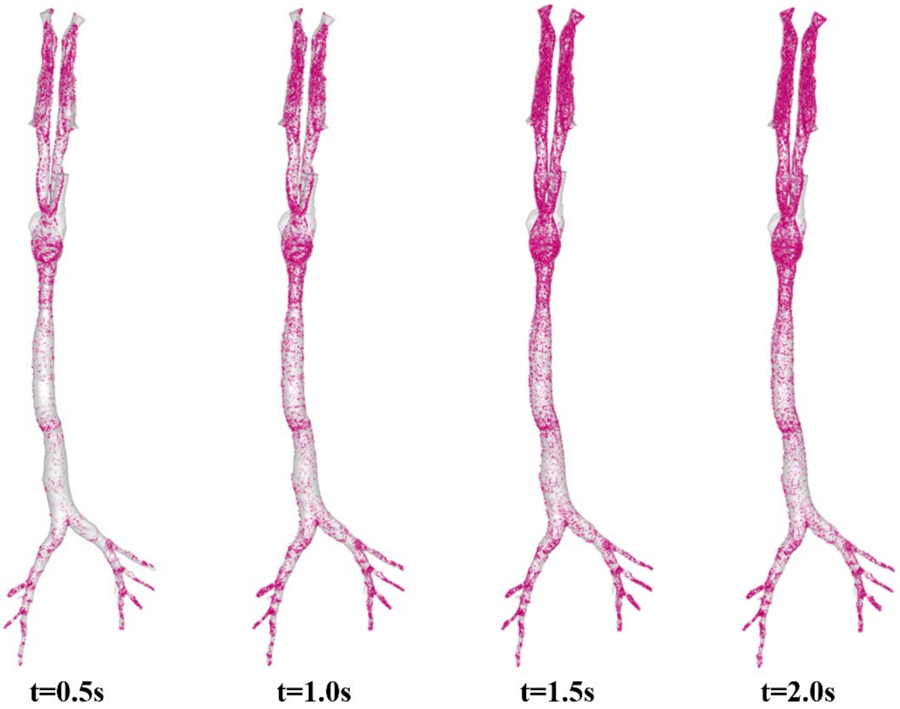

Figure S4 Aerosol deposition at characteristic moments: $t = 0.5$ s (peak inhalation point), $t = 1.0$ s (end of inspiratory phase), $t = 1.5$ s (peak expiratory point) and $t = 2.0$ s (end of a respiratory cycle). $Q = 17.0$ L/min, $T = 2$ s and aerosol dimeter $D = 2.5$ μm .

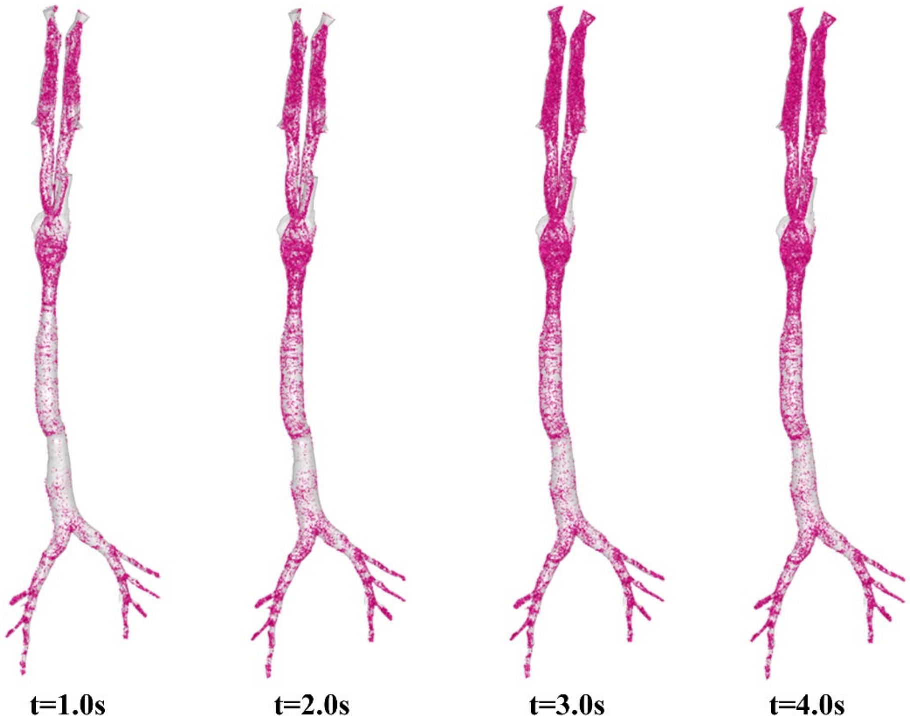

Figure S5 Aerosol deposition at characteristic moments: $t = 1.0$ s (peak inhalation point), $t = 2.0$ s (end of inspiratory phase), $t = 3.0$ s (peak expiratory point) and $t = 4.0$ s (end of a respiratory cycle). $Q = 17.0$ L/min, $T = 4$ s and aerosol dimeter $D = 2.5$ μm .

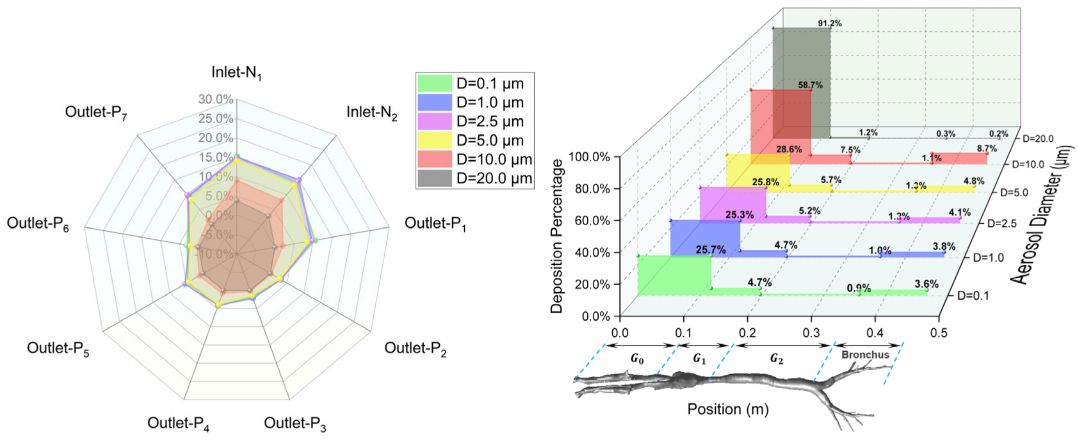

Figure S6 Regional (a) escape and (b) deposition fraction under $Q = 17.0$ L/min, $T = 2$ s. The naming of the inlets and outlets refers to Figure 3.

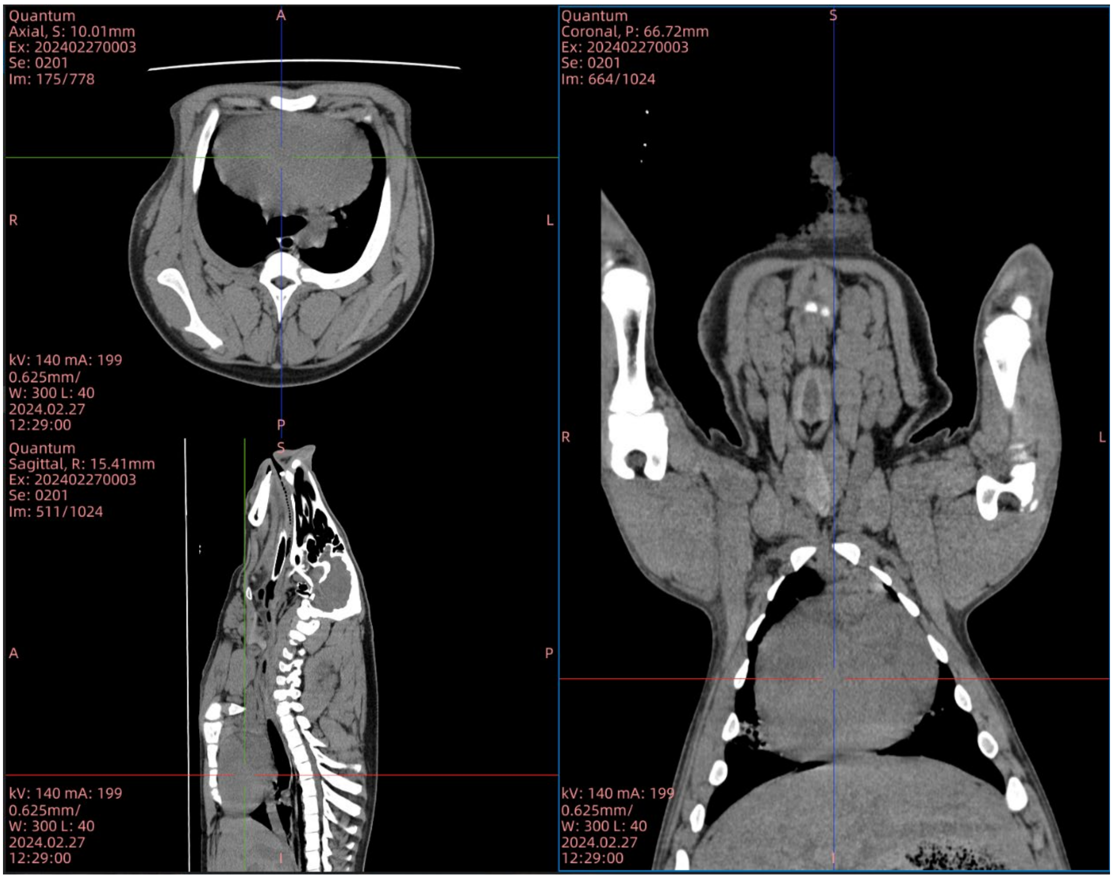

Figure S7 CT scan images of the pig showing the heart offset to the left. Note the left (L) and right (R) labels on the images.
